# Height associated variants demonstrate assortative mating in human populations

**DOI:** 10.1038/s41598-017-15864-x

**Published:** 2017-11-16

**Authors:** Xiaoyin Li, Susan Redline, Xiang Zhang, Scott Williams, Xiaofeng Zhu

**Affiliations:** 1 Department of Population and Quantitative Health Sciences, School of Medicine, Case Western Reserve University, Cleveland, OH 44106 USA; 2 Departments of Medicine, Brigham and Women’s Hospital and Beth Israel Deaconess Medical Center, Harvard Medical School, Boston, MA USA; 3 College of Information Sciences and Technology, The Pennsylvania State University, University Park, State College, PA USA

## Abstract

Understanding human mating patterns, which can affect population genetic structure, is important for correctly modeling populations and performing genetic association studies. Prior studies of assortative mating in humans focused on trait similarity among spouses and relatives via phenotypic correlations. Limited research has quantified the genetic consequences of assortative mating. The degree to which the non-random mating influences genetic architecture remains unclear. Here, we studied genetic variants associated with human height to assess the degree of height-related assortative mating in European-American and African-American populations. We compared the inbreeding coefficient estimated using known height associated variants with that calculated from frequency matched sets of random variants. We observed significantly higher inbreeding coefficients for the height associated variants than from frequency matched random variants (P < 0.05), demonstrating height-related assortative mating in both populations.

## Introduction

Human mate choice is relevant to a wide range of scientific disciplines, including biology, sociology, population genetics, evolutionary biology, and psychology^1–5^. Physical location, race, religion, ancestry, socioeconomic status (SES) and physical characteristics all influence mate choice^3,6–9^. Assortative mating, a phenomenon in which people choose mates with similar phenotypes to theirs in terms of physical traits and/or socio-cultural factors, is the most common deviation from random mating in Western societies^6,10,11^. Assortative mating studies have examined a wide array of factors for diverse purposes^11^. In general, age, education, race, religion and ethnic background show the strongest degree of assortative mating^3,11–19^. In addition to underlying biological traits, patterns of mate selection is often affected by the distribution of wealth and socioeconomic status, and taken together can impact on genetic structures of traits in a population if they are associated with genetic variation^11,16,20,21^.

From the population genetics perspective, assortative mating can affect heritability estimates, create correlations among traits that were initially unrelated and affect trait variance within and between families^22^. A key outcome of assortative mating is that it increases homozygosity of variants associated with traits that affect mate choice and causes an increase in genetic variance in a population and the corresponding trait variance, but does not change the allele frequencies unless the genetic variants are under differential selection^5^. When a trait forms a basis on which to select mates, it will inflate the estimated heritability for this trait based on parent-offspring studies^23,24^. If parental traits are correlated, then the offspring will have a higher probability of having the same alleles that affect the trait compared to their genomic backgrounds. In contrast, when estimating the heritability from twin studies, it is assumed that monozygotic (MZ) twins are genetically identical and share 100% of their genetic patterns, and dizygotic (DZ) twins share half of their genomes. Therefore, assortative mating does not affect trait correlation between MZ twins because MZ twins are genetically identical, but increases the correlation between DZ twins. As a result, assortative mating reduces the difference between MZ and DZ correlations, and which may lead to an underestimated heritability, if mating patterns are ignored^6,25,26^.

Assortative mating can create correlations between previously uncorrelated traits when these traits are involved in the mating selection preference^11,16^. Without accounting for assortative mating in genetic association studies, spurious associations may be observed for loci involved in the assortative mating process, and thus lead to an inflated false positive rate^27,28^. Another important aspect of assortative mating is that it increases the correlations between relatives for traits involved in mate choice, thereby increasing between-family variance^11^. Without properly modeling assortative mating, parameter estimates in association studies could be biased. Lastly, variants involved in assortative mating may be incorrectly eliminated from analyses because they violate Hardy-Weinberg equilibrium.

Among the traits that affect mate choice, e.g., education, SES, skin color, height is one that has been shown to be highly heritable, has a polygenic architecture, and is well studied genetically^5,10^. And because height has been associated with a range of health problems, such as cancers^29^, heart disease^30^, stroke^31^ and Alzheimer’s disease^32^, understanding how mate choice affects genotypes associating loci may help us to interpret results for these other traits as well. The estimated heritability of height is approximately 0.80 based on full-sib pair analysis^33^, but may be overestimated due to shared common environmental factors. Large GWAS studies identified common variants that together explain 50% to 60% of the heritability of adult height^34–36^. Genome wide association studies have identified about 700 variants associated with human height in individuals of European-ancestry^34,37^. These variants cumulatively explain approximately one fifth of the phenotypic variation in height and provide the most complete description of the genetic bases of a polygenic effect in humans. Although numerous height loci have been identified by GWA studies in Europeans, fewer have been reported in African-American populations, possibly because of smaller sample sizes and small estimated effect sizes of individual variants^38,39^. There is some debate whether spouse similarity for height can be explained by ancestry assortative mating^7,40^. Sebro *et al*.^3^ noted ancestry assortative mating in European Americans reflects a North-South European cline, which correlates with height. A recent study by the same group indicated that the height-related assortative mating is smaller than that for assortative mating by ancestry^41^. Thus, it is unclear whether assortative mating for height can be separated from the assortative mating for ancestry. Since assortative mating for height will only affect loci that contribute to height variation (and those in linkage disequilibrium with them), the genotype distributions of the identified height associated variants can be used to evaluate the evidence of assortative mating. In this study, we sought to quantify the genetic bases of height-related assortative mating by estimating the inbreeding coefficients of the height associated variants as compared to expectations for non-height associated loci. Simply, we tested the hypothesis that height associated variants have larger inbreeding coefficients than those for other loci in the genome. Results consistent with this hypothesis can provide complementary evidence that these variants are in fact height associated as it has previously been shown that deviations from Hardy Weinberg Equilibrium can provide independent evidence for association^42–45^.

## Results

### Spouse correlations of heights in CFS

The Cleveland Family Study (CFS) is an epidemiologic longitudinal study of participants who reside in Cleveland, Ohio. CFS recruited 645 European-Americans from 139 families and 652 African-Americans from 147 families^46^. We first calculated the height correlations between spouses. Table 1 shows the interclass spouse correlations in European-American and African-American cohorts in CFS. As expected, both European-Americans and African-Americans have a high height spouse correlation: r = 0.4 (P < 0.001) for European Americans and r = 0.24 (P = 0.14) for African Americans. The correlation in the African-American cohort was not significant, which was likely due to the smaller number of spouse-pairs (n = 39). Since ages of spouses may contribute to the height spouse correlation, we also calculated height residuals after adjusting for age in CFS founders. The height residual correlations between spouses are similar to those without adjusting for age (Table 1). The spouse height correlations provide support for height-related assortative mating in the European American cohort and modest support in the African American cohort.

**Table 1:** Spousal Correlations of height in the CFS cohorts.

Cohort	Correlation before adjusting for age			Correlation after adjusting for age			Number of spouse pairs
	Correlation	95% CI	P-value	Correlation	95% CI	P-value	
CFS European	0.40	(0.21,0.57)	1.3 × 10^−04^	0.38	(0.19,0.55)	2.8 × 10^−04^	85
CFS African	0.24	(−0.08,0.52)	0.14	0.14	(−0.19,0.44)	0.40	39

CFS–Cleveland Family Study.

CI–Confidence Interval.

### Genetic impact of height-related assortative mating

We estimated the inbreeding coefficients of height associating SNPs in the two European-American cohorts and five African-American cohorts by maximizing the likelihood in equations (2) and (3) (See Analytical Methods). For European-American populations, we obtained the 697 independent height associated SNPs from the European GWAS of the Genetic Investigation of Anthropometric Traits (GIANT) Consortium^34^. These 697 independent variants are located in 432 loci, and their corresponding genes are enriched in biological pathways for human skeletal growth. Among the 697 height-associated SNPs, 196 and 270 SNPs were directly genotyped in ARIC and CFS cohorts, respectively. An additional 315 and 325 SNPs could be replaced by proxy SNPs based on LD (*r* ^2^ > 0.9) derived using the 1000 G reference panel, which provides 511 and 595 height-associated SNPs for the two European-American cohorts, respectively (Table 2). Since height is a polygenic trait, we further selected the 2,500 and 5,000 independent SNPs with smallest P-values from the GWAS of the GIANT consortium^34^, respectively. We calculated the inbreeding coefficients using the 2,500 and 5,000 independent SNPs and compared these to frequency matched random SNPs in ARIC European cohort.

**Table 2:** Comparison of inbreeding coefficient estimated from height associated variants with randomly sampled frequency matched variants: single locus analysis.

Populations		Height-associated SNPs		Randomly sampled Frequency matched SNPs		P-value		Sample size
		Mean $\hat{f}\,$(sd)	# snp	Mean $\hat{f}\,$(sd)	# snp available for resampling	KS-test*	T-test	
*European American*	ARIC	−1.137 × 10^−03^ (1.7 × 10^−02^)	521	−3.296 × 10^–03^ (1.5 × 10^–02^)	68,423	4.18 × 10^−01^	6.14 × 10^−01^	6,787
		6.02 × 10^−04^ (1.4 × 10^−02^)	2,500	−2.125 × 10^−03^ (1.47 × 10^−02^)		1.65 × 10^−05^	3.74 × 10^−09^	
		6.5 × 10^−04^ (1.4 × 10^−02^)	5,000	−1.801 × 10^−03^ (1.46 × 10^−02^)		8.08 × 10^−08^	3.41 × 10^−15^	
	CFS	4.173 × 10^–03^ (7.6 × 10^–02^)	595	−4.917 × 10^–03^ (7.6 × 10^–02^)	64,749	1.23 × 10^–09^	3.83 × 10^–03^	171
*African American*	CARDIA	9.764 × 10^–03^ (6.6 × 10^–02^)	158	−2.21 × 10^–03^ (4.0 × 10^–02^)	139,703	1.17 × 10^–02^	2.31 × 10^–02^	828
	MESA	1.276 × 10^–02^ (9.4 × 10^–02^)	168	2.445 × 10^–04^ (3.1 × 10^–02^)	141,317	1.43 × 10^–02^	2.96 × 10^–02^	1,147
	JHS	1.22 × 10^–02^ (6.7 × 10^–02^)	165	−6.388 × 10^–04^ (3.4 × 10^–02^)	141,484	1.80 × 10^–03^	1.48 × 10^–02^	941
	CFS	2.414 × 10^–02^ (1.16 × 10^–02^)	166	−9.103 × 10^–03^ (9.0 × 10^–02^)	119,600	7.51 × 10^–07^	2.97 × 10^–04^	121
	ARIC	8.4687 × 10^–03^ (6.1 × 10^–02^)	159	−1.796 × 10^–03^ (2.9 × 10^–02^)	139,239	3.56 × 10^–01^	3.60 × 10^–02^	1,504

^*^Kolmogorov–Smirnov test.

sd–standard deviation.

ARIC–Atherosclerosis Risk in Communities; CFS - Cleveland Family Study; CARDIA - Coronary Artery Risk Development in Young Adults; JHS - Jackson Heart Study.

For African-American cohorts, we included the top 169 SNPs (*P* < 5 × 10^−5^) identified from the GWAS of the Women’s Health Initiative (WHI)^39^ for the height-related assortative mating analysis. The number of SNPs genotyped in African-American cohorts range from 158 to 168 (Table 2).

### Assortative mating analysis at a single locus

**Figure 1:**
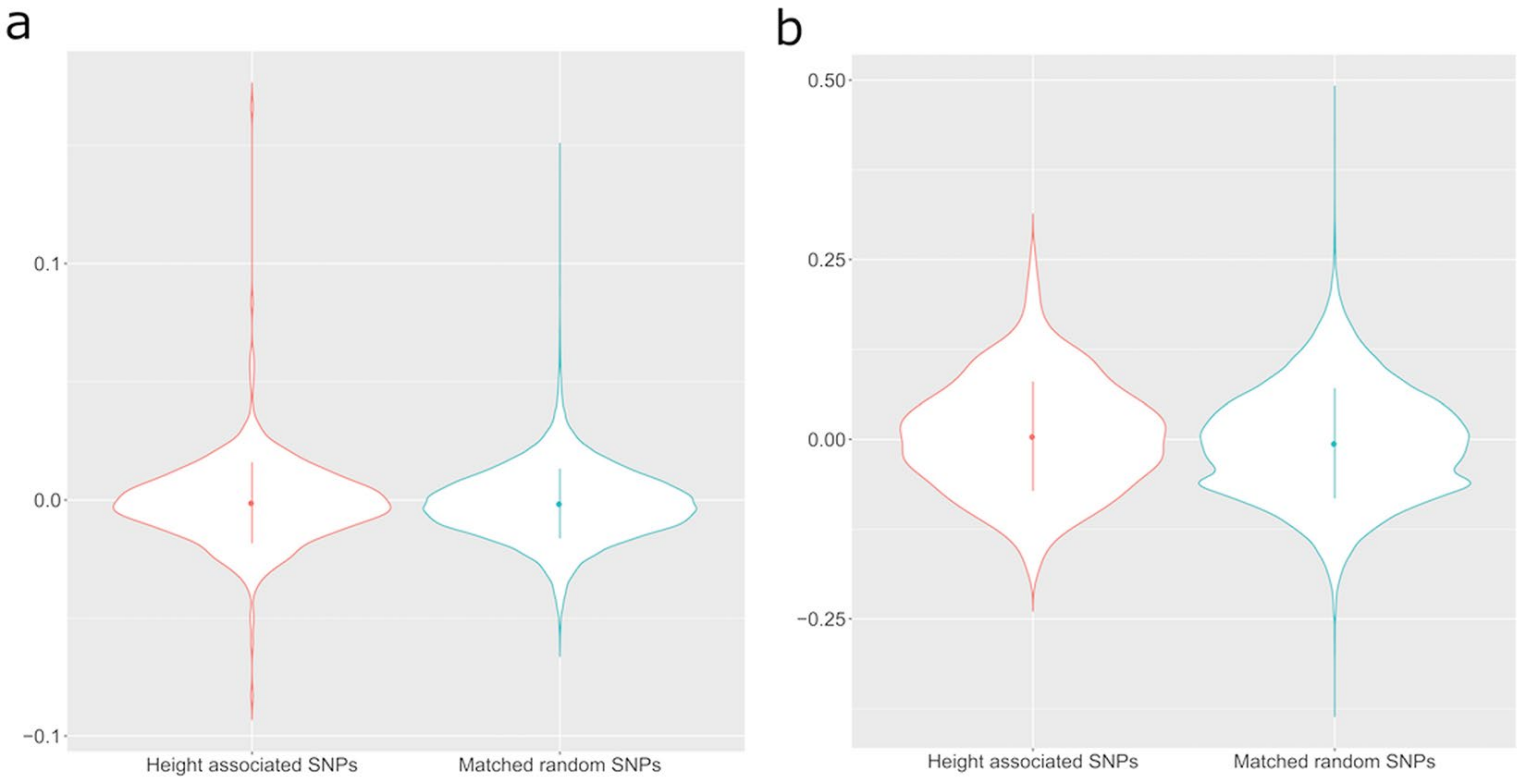
The violin plots of the inbreeding coefficient at single locus level for European-American cohorts. Red represents height associated loci and teal represents frequency matched random SNPs. (**a**) ARIC; (**b**) CFS.

**Figure 2:**
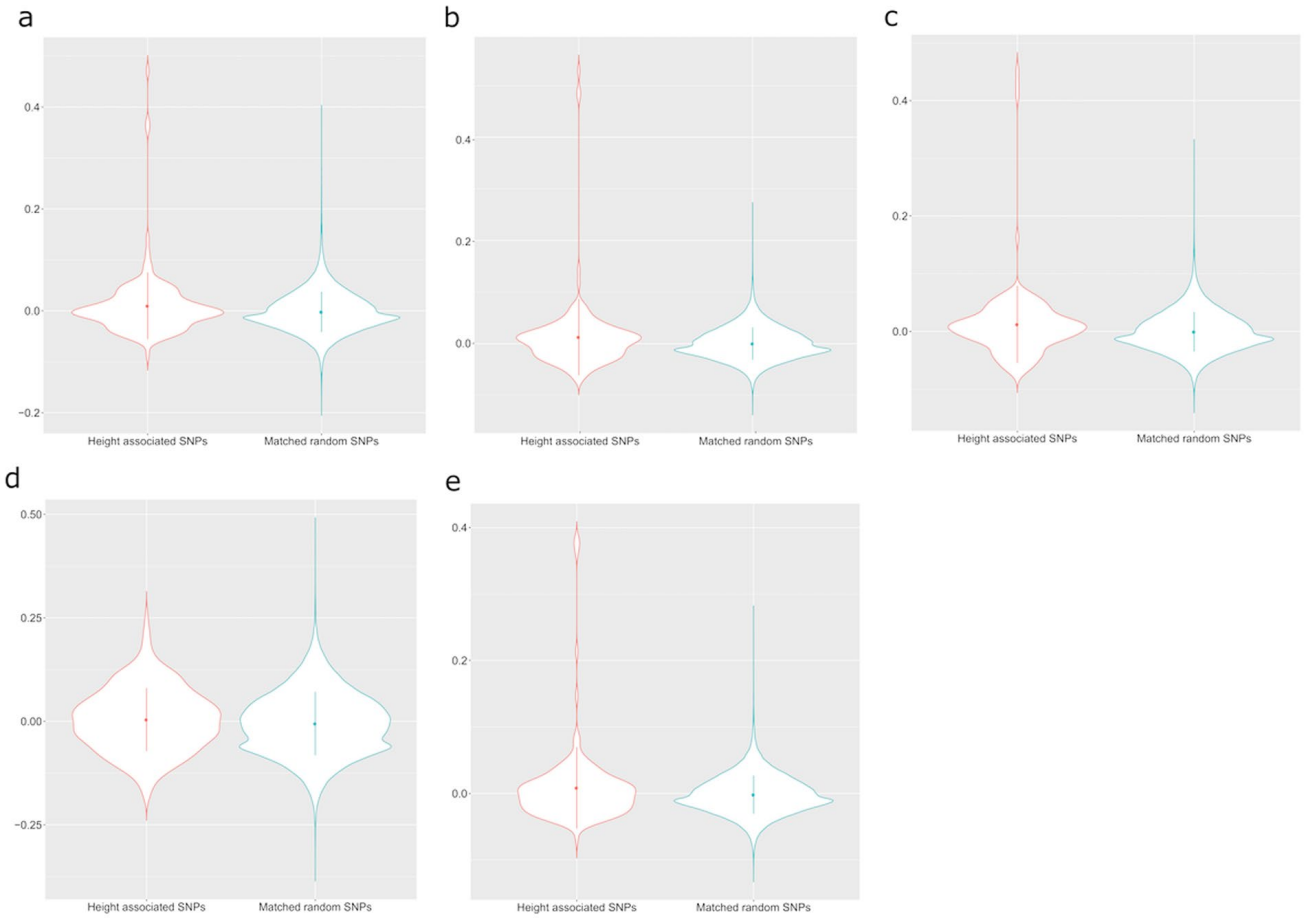
The violin plots of the inbreeding coefficient at single locus level for African-American cohorts. Red represents height associated loci and teal represents frequency matched random SNPs. (**a**) CARDIA; (**b**) MESA; (**c**) JHS; (**d**) CFS; (**e**) ARIC.

Average inbreeding coefficients in the two European-American and five African-American cohorts, using the height associated SNPs, were calculated and compared to frequency matched randomly selected SNPs from the same cohorts, as well as to the whole genome. (Table 2 and Supplementary Table S2) (equation (2) in Analytical Methods). In the two European-American cohorts, the average inbreeding coefficients for height associated SNPs are −1.137 × 10^−3^ and 4.173 × 10^−3^ for ARIC and CFS, respectively. The average of single inbreeding coefficients for height associated SNPs ranges from 8.4687 × 10^−3^ to 2.414 × 10^−2^ in five African-American cohorts. We randomly selected the same number of independent SNPs with minor allele frequencies matched to the height associated SNPs for each cohort and estimated their corresponding inbreeding coefficients. We observed significant differences for the inbreeding coefficients between the height associated SNPs and the random set of SNPs in all the cohorts except the ARIC European cohort (P-value < 0.05 for all cohorts except for ARIC European cohort, Table 2), with the height associated SNPs always having higher inbreeding coefficients. Although not statistically significant, the trend in the ARIC European cohort was the same as for the other cohorts. The violin plots also show the distribution difference between inbreeding coefficients estimated using height associated variants and randomly matched variants across the genome except ARIC European cohort (Figs 1 and 2). Thus, the genetic results provide evidence of assortative mating for height associated SNPs in all cohorts except for the ARIC European one.

We observed negative average inbreeding coefficients for randomly selected SNPs in most of our studied cohorts (Table 2), although average inbreeding coefficients were close to 0. We also observed a negative average inbreeding coefficient for height associated SNPs in the ARIC European cohort. Since height is a polygenic trait, we selected the independent 2,500 and 5,000 SNPs with the smallest P-values in the height GWAS of the GIANT consortium^34^, respectively. We repeated the analysis using these 2,500 and 5,000 SNPs in the ARIC European cohort. We observed that the average inbreeding coefficients became more positive as more top height-associated SNPs were included, with the average inbreeding coefficients changing to 6.02 × 10^−4^ and 6.5 × 10^−4^ for the top 2,500 and 5,000 SNPs, respectively, among ARIC European Americans (Table 2), as compared to a negative value for the GWAS significant SNPs only. The difference became more significant when comparing with frequency matched random SNPs (P < 2 × 10^−5^ for the 2,500 SNPs and P < 9 × 10^−8^ for the 5,000 SNPs for all conducted tests). We calculated the correlation between effect size and inbreeding coefficient using the 521 genome wide significant SNPs and their corresponding inbreeding coefficients. We did not observe a significant correlation (r = −0.02, p = 0.545). Our result indicates that inbreeding coefficient is independent of the effect size of height associated variants, and the estimated average inbreeding coefficient is likely underestimated when only top of height associated markers are used for analysis.

**Figure 3:**
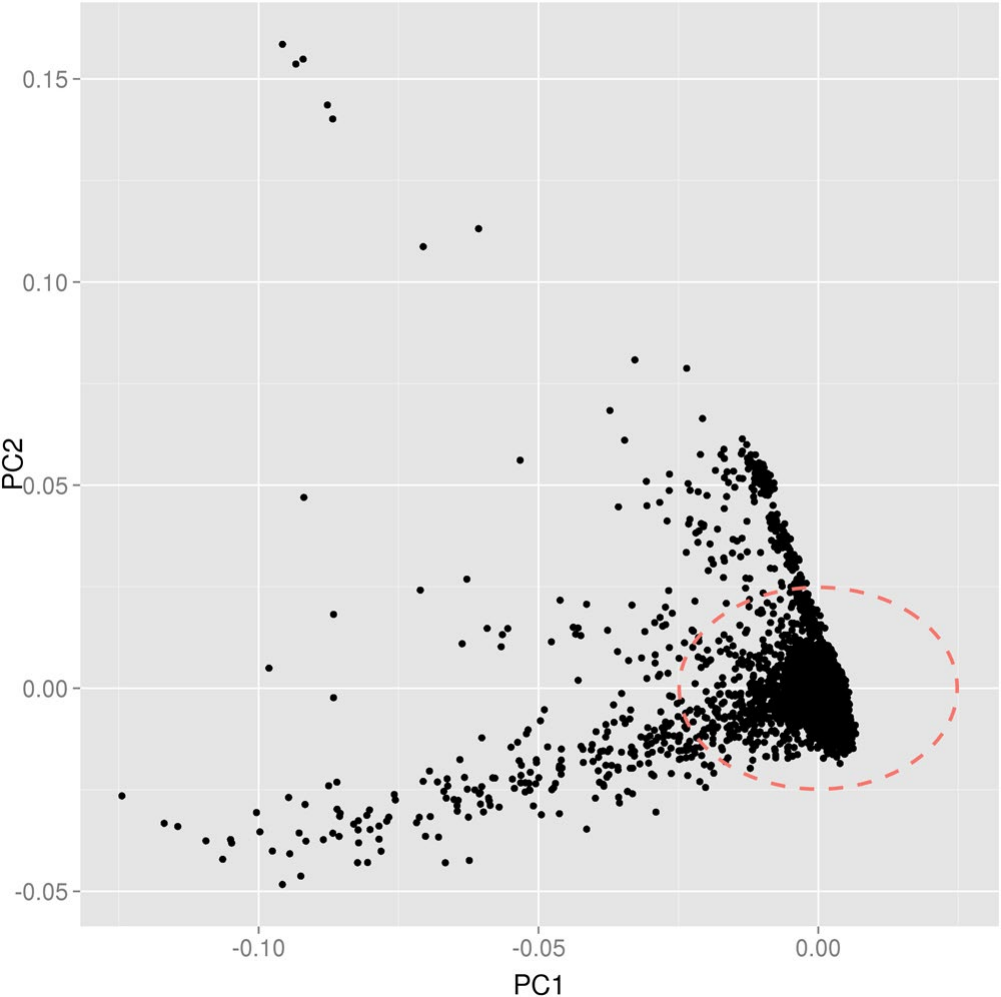
Plot of the first two principal components for 6,787 unrelated ARIC European subjects.

As population structure will impact inbreeding coefficient estimates, we examined the population structure in the ARIC European cohort using principal component (PC) analysis^9,47,48^. The North-South European admixture can be clearly observed (Fig. 3). We then excluded the outliers identified using the first two PCs (Fig. 3) and calculated inbreeding coefficients again. The estimated inbreeding coefficients are consistent with those obtained from all samples, which ranges from −9.24 × 10^−4^ to 6.63 × 10^−4^ using a variable number of variants. Again we observed a significant shift of inbreeding coefficients using height associated variants as compared to randomly selected frequency matched variants (P < 0.05 for top 2,500 SNPs and 5,000 SNPs) (Supplementary Table S1).

### Assortative mating analysis with multiple loci

**Figure 4:**
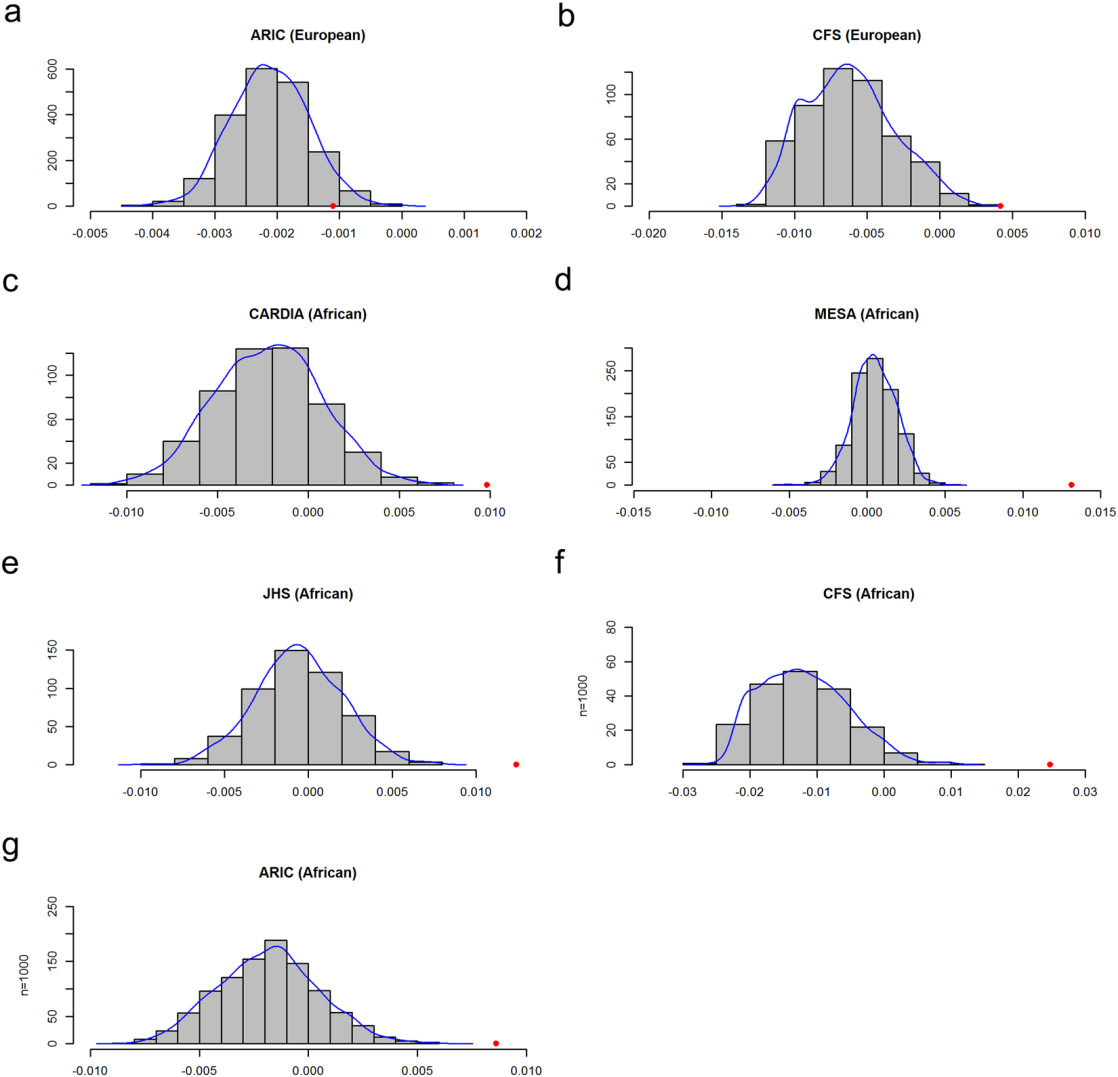
The histogram of the inbreeding coefficient using multiple loci for both European-American and African-American cohorts. Distributions were based on 1,000 resampling. The observed inbreeding coefficient for height associated SNPs are marked in red points. (**a**) ARIC (European); (**b**) CFS (European); (**c**) CARDIA (African); (**d**) MESA (African); (**e**) JHS(African); (**f**) CFS (African); (**g**) ARIC (African).

We further calculated the inbreeding coefficient using all of the height associated variants using equation (3) in Analytical Methods. Table 3 lists the inbreeding coefficients estimated from all height-associated variants in each cohort. The estimated inbreeding coefficients are −1.1 × 10^−3^ and 4.2 × 10^−3^ for ARIC European and CFS European, respectively. For the five African-American cohorts, the estimated inbreeding coefficients range from 8.62 × 10^−3^ to 2.477 × 10^−2^. The estimated inbreeding coefficients using all height-associated loci are approximately equivalent to the average of inbreeding coefficient for the single locus analysis, as expected. We observed that the inbreeding coefficients estimated using height associated variants fall in the right tails of the inbreeding coefficient distributions calculated using randomly sampled allele frequency matched SNPs (see Analytical Methods) for all the cohorts, and they are all statistically significant (Fig. 4, Table 3, $P < 0.05$). Thus, our results are consistent with assortative mating by height driving increased homozygosity of SNPs associated with height in both European-American and African-American cohorts. As expected, when including more of the most associated SNPs in the ARIC European cohort, the inbreeding coefficients become positive and remain statistically significant (Table 3, $P < 0.05$), supporting the polygenic basis of human height.

**Table 3:** Comparison of inbreeding coefficient estimated from height associated variants and lipids associated variants with randomly sampled frequency matched variants: multiple loci analysis.

Trait	Population		$\widehat{{{\boldsymbol{f}}}_{{\boldsymbol{M}}}}\,$(sd)	average $\widehat{{{\boldsymbol{f}}}_{{\boldsymbol{M}}}}$ of frequency matched variants (sd)	P-value*	# of SNPs analyzed
Height	*European American*	ARIC	−1.10 × 10^–03^ (5.32 × 10^–04^)	−2.19 × 10^–03^ (6.16 × 10^–04^)	0.027	521
			6.164 × 10^–04^ (2.561 × 10^–04^)	−2.15 × 10^–03^ (3.21 × 10^–04^)	0.001	2,500
			6.476 × 10^–04^ (1.889 × 10^–04^)	−2.09 × 10^–03^ (6.16 × 10^–04^)	0.001	5,000
		CFS	4.20 × 10^–03^ (3.16 × 10^–03^)	−6.19 × 10^–03^ (3.01 × 10^–03^)	0.025	595
	*African American*	CARDIA	9.847 × 10^–03^ (2.83 × 10^–03^)	−2.211 × 10^–03^ (2.94 × 10^–03^)	0.001	158
		MESA	1.313 × 10^–02^ (2.36 × 10^–03^)	4.749 × 10^–04^ (2.48 × 10^–03^)	0.001	168
		JHS	1.242 × 10^–02^ (2.62 × 10^–03^)	−5.618 × 10^–04^ (2.60 × 10^–03^)	0.001	165
		CFS	2.477 × 10^–02^ (7.38 × 10^–03^)	−7.483 × 10^–03^ (4.56 × 10^–03^)	0.001	166
		ARIC	8.622 × 10^–03^ (2.10 × 10^–03^)	−1.825 × 10^–03^ (2.29 × 10^–03^)	0.001	159
Lipids	*European American*	ARIC	−1.167 × 10^–03^ (9.87 × 10^–04^)	−2.21 × 10^–03^ (1.15 × 10^–03^)	0.29	152
		CFS	3.992 × 10^–03^ (8.806 × 10^–03^)	−5.28 × 10^–03^ (8.40 × 10^–03^)	0.152	157

^*^P-value is comparing $\widehat{{f}_{M}}$ using height variants and randomly sampled frequency matched variants.

sd–standard deviation.

ARIC–Atherosclerosis Risk in Communities; CFS - Cleveland Family Study; CARDIA - Coronary Artery Risk Development in Young Adults; JHS–Jackson Heart Study.

To test whether any trait associated SNPs will be affected by assortative mating, we repeated the analyses using blood lipids associated variants obtained from the Global Lipids Genetics Consortium^49^ in European populations. The estimated inbreeding coefficients for lipids associated SNPs were not statistically significant for all analyses (Table 3), indicating that there is no or much weaker assortative mating for blood lipids than for height.

### Linkage Disequilibrium Analysis

We further assessed assortative mating for height by regressing pairwise linkage disequilibrium (LD) score on the products of the first two PC loadings and the product of effect sizes of height associated variants in the ARIC European cohort, a method demonstrated to be robust with respect to population structure^5,22^. We calculated the unstandardized LD parameter D^16,50^ for height associated SNPs located on different chromosomes and their corresponding PC loadings for PC1 and 2 in the ARIC European cohort. Using linear regression, we obtained the effect sizes for these height variants. We then regressed the D values for a pair of height variants on the products of height effect sizes and the products of PC-loadings for each pair of SNPs^41^. We observed significance for both height effect size products (P = 9.62 × 10^−12^) and PC-loading products (P = 6.33 × 10^−56^ for PC1 and P = 5.06 × 10^−41^ for PC2) (Table 4), providing further evidence for strong assortative mating by height that was independent of ancestry and population structure.

**Table 4:** Regression analysis of linkage disequilibrium parameter D on the product of height effect sizes and PC-loadings for unlinked SNPs in ARIC European cohort.

	Estimate (sd)	T-value	P-value
*Product of height effect sizes*	1.186 × 10^−03^ (1.741 × 10^−04^)	6.813	9.62 × 10^−12^
*Product of PC1-loading*	1.069 × 10^−04^ (6.782 × 10^−06^)	15.766	6.33 × 10^−56^
*Product of PC2-loading*	8.777 × 10^−05^ (6.540 × 10^−06^)	13.420	5.06 × 10^−41^

sd–standard deviation.

## Discussion

In this study, we examined assortative mating for height, using both phenotype and genotype data. Estimates of assortative mating based on spousal correlations was consistent with the literature^6,8,11,20,51^, with estimates of correlation between spouse-pairs ranging from 0.24 to 0.4. We observed that the estimated inbreeding coefficients for height associated variants were consistently larger than that for frequency matched random markers using either single or multiple locus analyses in both European Americans and African Americans. Since assortative mating can be affected by socio-demographic factors, Laurent *et al*.^4^ suggested to use the genome wide distribution as a control. We estimated the inbreeding coefficients across the genome in the studied cohorts (Supplementary Table S2 and Supplementary Figs S1–S3); the estimated inbreeding coefficients for height associated variants were consistently larger than that based on genome wide estimates. Assortative mating for height was also independent of ancestry as determined by regressing pairwise linkage disequilibrium (LD) score on the products of the first two PC loadings and the product of effect sizes of height associated variants in the ARIC European cohort (Table 4). Thus, our results show that genetic variants associated with height exhibit significant inbreeding coefficients as predicted by our hypothesis. These results clearly demonstrate the genetic effects of phenotype-based mating in humans.

Although assortative mating for height has been reported^10,11,18,25^, it was not clear whether assortative for height could be explained by ancestry assortative mating or population structure.^7,40^ Nor did prior studies estimate how strong the height-related assortative mating was after controlling population structure^41^. Since population structure should impact genotype distributions equally across the genome as long as the assessed variants are not under selection, our results show trait specific effects of mating behavior by comparing the inbreeding coefficients estimated using height associated variants with a frequency matched random variants. Since most genetic variants are neutral or nearly neutral our comparison should be representative of random mating across the genome^52^. Additionally, genetic variants with large fitness are generally rare or low frequency and we removed all the variants with MAF <0.01 to reduce the potential bias due to selection pressure. Finally, selection may also cause departure from HWE and such variants were also excluded. Therefore, our observations of larger inbreeding coefficients of height associated variants than that of random frequency matched variants most likely reflects assortative mating for height. The result is also consistent with that from regression analysis of pairwise linkage disequilibrium (LD) score on the products of the first two PC loadings and the product of effect sizes of height associated variants in the ARIC European cohort. We observed significant association between LD and height effect size after adjusting for the PC loadings of the first two PCs (Table 4). Sebro *et al*.^41^ using the same analysis in Framingham Heart Study only observed strong assortative mating for ancestry, but not height, possibly due to relatively small sample size and small number of height associated markers used in their analyses.

Another possible cause of increased inbreeding coefficients in our analyses, is that GWAS significant SNPs may have different characteristics than random SNPs from across the genome. If this is the case, our evidence for assortative mating for height may reflect a general characteristic for GWAS significant SNPs in general. To assess this possibility, we performed the same analysis with the GWAS significant SNPs associated with blood lipids, and no significant inbreeding coefficient inflation was observed, although SNPs associated with blood lipids did show a trend towards assortative mating (Table 3). We are not clear what causes this tendency. However, it is possible that the tendency may reflect the correlation between growth in height and blood lipids^53^. This result indicates that GWAS associating SNPs, in general, do not inflate inbreeding coefficients, further supporting our main conclusions.

The inbreeding coefficient for height associated SNPs was negative in the multiple locus analysis in the ARIC European cohort, although the results demonstrated significantly larger inbreeding coefficients as compared to the randomly selected SNPs (Table 3 and Fig. 4). This was an unexpected observation. However, multiple reasons can lead to negative inbreeding coefficient estimates. (1) When sample size is finite, population genetics theory indicates that the heterozygote frequencies are increased by 1/(2N-1), where N is population effective size under random mating (Crow and Kimura, Introduction to Population Genetics Theory^5^, page 55), and this may result in negative average inbreeding coefficient estimates. (2) In F_1_ populations, the homozygote frequency will decrease by an amount of the variance of frequency among subpopulations (Crow and Kimura, Introduction to Population Genetics Theory^5^, page 54). In admixed populations, there can be many subjects whose parents are from different ancestries, even if defined as European. For example, the ARIC cohort probably has numerous samples where one parent was from Northern Europe and the other from Southern Europe (Fig. 3). When we assessed only individuals with less admixture as identified with the first two PCs (Fig. 3), the estimated inbreeding coefficients shifted to being less negative, although the differences were small (Supplementary Table S1). Similar population admixture occurs in the other cohorts (Supplementary Fig. S4). Hence, as predicted population admixture leads to lower inbreeding coefficients via increased heterozygosity across all loci, whether they have a phenotypic impact or not. (3) We estimated the pairwise kinship coefficient among individuals and excluded one individual of each pair with an estimated kinship coefficient >0.025, which will bias average inbreeding coefficient estimates in a negative direction.

To further investigate the negative inbreeding coefficients, we analyzed the ~2,500 and ~5,000 most significant height associated SNPs from the GIANT height genome wide association study. The estimated inbreeding coefficients became more positive on average with an increasing number of height-associated SNPs. Increasing the number of marginally significant height SNPs in the estimates of inbreeding coefficients increased the difference with respect to the random SNPs (P < 2 × 10^−5^ for top 2,500 SNPs and P < 9 × 10^−8^ for top 5,000 SNPs), further providing evidence of height-based assortative mating in the ARIC European cohort (Tables 2 and 3). As height is a highly heritable trait with an estimated heritability of 80% and a very large number of genetic variants (as many as 100,000 variants^54^) that may contribute to its variation^33^, it is possible that some of our randomly selected SNPs are actually associated with height. If this is the case, then our resampling analyses are conservative in testing for assortative mating. Nonetheless, we found evidence for height related assortative mating in all studied cohorts. It should be noted that our method cannot differentiate active assortative mating from passive assortative mating, i.e., that related to social or geographical homogamy.

We noted that the inbreeding coefficients estimated from either single variant or multiple variants are small and may not have substantial effect to HWE estimates. One reason is that we eliminated all variants with substantial evidence of the departure from HWE via QCs. The second reason is that there are a large number of height variants. When assortative mating involves a large number of variants, it will be less likely to affect HW deviations^37,55^. However, we still observed consistent larger inbreeding coefficients for the height associated variants than for a random set of variants.

We observed that the minor allele frequencies after LD pruning have a U-shape distribution with an excess of variants with intermediary frequencies^56,57^ (Supplementary Figs S5 and S6). The enrichment of higher minor allele frequency SNPs was caused by the LD pruning procedure as implemented in PLINK that keeps the SNPs with higher minor allele frequencies when performing LD pruning^58^. However, the inbreeding coefficient does not depend on allele frequency. To examine whether the allele frequency spectrums affect our result, we redid the LD pruning by selecting the retained SNPs at random. The inbreeding coefficients from height-associated SNPs compared to the randomly selected frequency matched SNPs from the LD pruning was not affected by MAF. We observed the same assortative mating signature for height. (Supplementary Table S3 and Supplementary Figs S5, S6). Our results suggested that the LD pruning process did not affect our conclusions.

It is possible that the estimation of inbreeding coefficient may be biased if a disease is associated with height and study cohorts were disease oriented. However, our study cohorts are population based samples. We only included adults and adult height is less impacted by disease. Therefore, our conclusion of assortative mating for height should not be affected even if our study cohorts include some unhealthy subjects.

In summary, our results confirmed previous reports of assortative mating by height in both European-American and African-American populations, but in contrast to studies of just assessing phenotypic correlations, we were able to demonstrate measurable genetic effects of this mating behavior. Our results indicate that mate choice with respect to height affects genotypes at loci associating with height, providing independent evidence of the veracity of these variants as associating with height. However, it is still not clear how much impact non-random mating has on genetic association studies that typically assume random mating. Our results indicate that care will need to be taken when assessing variants for association with respect to assumptions of random mating and levels of heritability as previous work has shown that heritability estimates will be inflated when the phenotypic correlation reflects genotypic correlation^59^. Statistical approaches considering non-random mating may be helpful in genetic association analysis, heritability estimation or interpretation of results.

## Materials and Methods

The study used existing datasets, including CFS phenotype and genotype data and CARe genotype data. The CFS phenotype data were analyzed anonymously at Case Western Reserve University. The CFS study was approved by Partners Human Research Committee with the proposal number 2011D001860. Our study has been approved by Case Western Reserve University Institutional Review Board (IRB-2013-525). The genotype data from the Candidate Gene Association Resource (CARe) consortium were downloaded from the dbGaP.

### Cohort description

The European cohorts included Cleveland Family Study (CFS) and Atherosclerosis Risk in Communities (ARIC). The CFS is a family-based longitudinal study starting in 1990 comprised of index cases with laboratory diagnosed sleep apnea, their family members, and neighborhood control families^60,61^. Four examinations over 16 years included measurements of sleep apnea, anthropometry, and other related phenotypes, as detailed previously^60,61^. The CFS (dbGaP phs000284.v1.p1) includes 645 European Americans in 139 families who were genotyped on the OmniChip 2.5 M array. The ARIC data were downloaded from dbGaP database (dbGaP phs000090.v1.p1). The ARIC study, sponsored by the National Heart, Lung and Blood Institute (NHLBI), is a prospective epidemiologic study designed to investigate the etiology and natural history of atherosclerosis, the etiology of clinical atherosclerotic diseases, and variation in cardiovascular risk factors, medical care and disease by race, gender, location, and date. It includes 9,707 independent subjects genotyped by Affymetrix 6.0 array.

The African-American samples are from the Candidate Gene Association Resource (CARe) consortium^62^. CARe has assembled samples from 9 community-based cohorts representing four ethnic groups: European-American, African-American, Hispanic, or Chinese-American, as described in detail^62^. The African-American samples for our assortative mating analysis were obtained from five CARe cohorts: Atherosclerosis Risk in Communities (ARIC: dbGaP phs000280.v1.p1), Coronary Artery Risk Development in Young Adults (CARDIA: dbGaP phs000285.v2.p2), Cleveland Family Study (CFS: dbGaP phs000284.v1.p1), Jackson Heart Study (JHS: dbGaP phs000286.v1.p1), Multi-Ethnic Study of Atherosclerosis (MESA: dbGaP phs000283.v1.p1), a detailed description of each cohort can be found in^63^. Genotyping for those cohorts was performed with Affymetrix 6.0 array.

### Quality Controls

All data quality controls (QCs) were performed for each cohort separately, and only autosomal loci were used. We selected the height associated variants from the most recent GWAS^34,39^ in both European-American and African-American populations to determine the degree of height-based assortative mating. The remaining SNPs were considered for use in a comparison group. For the set of non-height associated loci, we excluded SNPs in each individual dataset that had either a call rate (CR) < 0.95, a minor allele frequency (MAF) < 0.01 or $P < 5e-7$ from a Hardy-Weinberg equilibrium test, using software PLINK^58^. Individuals with a missing genotype rate > 0.1 were also removed. After QCs, ~600,000 markers remained in European-American cohorts for analysis. For the five African-American cohorts, ~800,000 markers passed QCs. Since our analysis assumed all markers are independent, we pruned SNPs using PLINK^58^ (r^2^ < 0.1). After pruning, the number of SNPs in analysis were between 68,453 and 65,069 for ARIC and CFS European-American cohorts, and between 119,725 and 189,966 SNPs for African-American cohorts, respectively. The minor allele frequency distributions for height associated variants and all variants across the genome are shown in Supplementary Figs S5 and S6.

To ensure the estimated inbreeding coefficients were not confounded by the related family members, we selected unrelated founders for the family-based cohorts (CFS and JHS). To avoid cryptic relatedness, we estimated the pairwise kinship coefficient among individuals using genome wide SNPs in each cohort by software GCTA^64^ and excluded one individual of each pair with an estimated kinship coefficient >0.025. The final sample sizes were presented in Table 2. For admixed populations, it may be more accurate to use REAP^65^ that requires allele frequency distributions in ancestral populations, which were not available for our European American cohorts. Since the estimated kinship coefficients from GCTA and REAP are highly correlated and we only estimated kinship coefficients, it should have little effect for the inbreeding coefficient estimates. Therefore, the difference in method should not affect our conclusions.

### Analytical Methods

Assume that a marker with two alleles A and a, and the corresponding three genotypes are aa, Aa, or AA, with allele frequency $\,f(A)=p$ and $\,f(a)=q$ subject to the constraint $p+q=1$. If a population displays random mating, the expected genotype frequencies follow the Hardy-Weinberg law with the genotype frequencies $\,f(AA)={p}^{2}$, $\,f(Aa)=2pq$ and $\,f(aa)={q}^{2}$ for AA homozygotes, Aa heterozygotes and aa homozygotes, respectively. The Hardy-Weinberg principle describes a panmictic population with no mutation, migrations or selection. Either inbreeding or assortative mating will lead to Hardy-Weinberg disequilibrium, although inbreeding will affect all genetic variants while assortative mating will only involve loci related to traits associated with phenotypes affecting mate selection^5^. In either case, the genotype frequencies can be written as:

1 $$\begin{array}{c}AA:{p}^{2}\,(1-f)+pf\\ Aa:2pq\,(1-f)\\ aa:{q}^{2}\,(1-f)+qf\end{array}$$

where $f$ is the inbreeding coefficient^5^. Both inbreeding and assortative mating will increase homozygote and decrease heterozygote frequencies. An inbreeding coefficient ranges between $0$ and 1. In the extreme case of self-fertilization, the inbreeding coefficient is 1. When the frequency of heterozygotes equals the HW expectation then the inbreeding coefficient is 0.

#### Assortative mating at a single locus

Assuming ${n}_{2}$ and ${n}_{0}$ are the observed number of homozygotes, ${n}_{1}$ the observed number of heterozygotes. To estimate the inbreeding coefficient $f$ at a single locus, we applied the maximum likelihood method^66^ which maximizing the following log likelihood (logl):

2 $$logl(f,p,q)={n}_{2}log({p}^{2}\,(1-f)+pf)+{n}_{1}\,\mathrm{log}(2pq\,(1-f))+{n}_{0}\,\mathrm{log}(\,{q}^{2}\,(1-f)+qf)$$

Note that the allele frequency is unaffected by inbreeding and assortative mating. Thus, we can maximize the inbreeding coefficient using an estimated allele frequency $p$ and $q=1-p$.

To test whether assortative mating exists in each cohort, we calculated the inbreeding coefficients when estimated using resampled frequency matched variants across the genome after excluding the height associated loci. Since these resampled SNPs are less likely to be height associated, the distribution of estimated inbreeding coefficient from resampling should reflect the distribution without assortative mating on this trait. We further performed a two sample T-test as well as a Kolmogorov–Smirnov test (KS-test) to compare the height-associated SNPs with the randomly selected frequency matched SNPs.

#### Assortative mating with multiple loci

We extended the maximum likelihood method to estimate the inbreeding coefficient at a set of height-associated loci. Consider a set of $M\,\,$independent SNPs, the inbreeding coefficient at multiple loci is denoted by ${f}_{M}$. For the ${i}^{th}$ SNP, the minor allele frequency is assumed to be $\,{p}_{i}$, and $\,{n}_{0i}$ and ${n}_{2i}$ denote the observed number of homozygotes, ${n}_{1i}$ denote the observed number of heterozygotes. Then the likelihood function for the $M\,\,$independent SNPs is

3 $$l({f}_{M},{p}_{1},\cdots {p}_{M})=\prod _{i=1}^{M}{[{p}_{i}^{2}(1-{f}_{M})+{p}_{i}{f}_{M}]}^{{n}_{2i}}\times [2{p}_{i}(1-{p}_{i})\,(1-{f}_{M})){]}^{{n}_{1i}}\,\times {[{(1-{p}_{i})}^{2}(1-{f}_{M})+(1-{p}_{i}){f}_{M}]}^{{n}_{0i}}$$

Here we assume that the inbreeding coefficient is the same for the $M\,\,$independent SNPs, and therefore, the estimated inbreeding coefficient $\widehat{{f}_{M}}$ can be interpreted as the common inbreeding coefficient for the $M\,\,$independent SNPs. Using the same considerations as for a single variant, the allele frequency for each SNP does not change for either inbreeding or assortative mating and can be estimated independently. The inbreeding coefficient $\widehat{{f}_{M}}$ can then be estimated using computational optimizations.

When a set of SNPs contributes to trait variation involved in assortative mating, the estimated inbreeding coefficient $\widehat{{f}_{M}}$ from equation (3) will be affected by both inbreeding (genome wide effects) and assortative mating (locus specific). Population substructure is also a confounder for estimating the inbreeding coefficient, but should affect all loci similarly. We estimate the empirical distribution of $\widehat{{f}_{M}}$ under the null hypothesis that there is no height associated  assortative mating, but possibly population structure or cryptic relatedness. To obtain a distribution of $\widehat{{f}_{M}}$ under the null of no assortative mating, we applied a resampling procedure. In each resampling, we randomly sample the same number, $M$, of independent SNPs with matched allele frequencies from the genome and calculate the inbreeding coefficient $\widehat{{f}_{M}}$. This resampling procedure was repeated 1,000 times to obtain a null distribution of $\widehat{{f}_{M}}$. Since most of genome wide variants either do not contribute to the height variation or have effect sizes that are small, the estimated $\widehat{{f}_{M}}$ is the approximate distribution under the null hypothesis of absence of assortative mating. The test for height-related assortative mating can be obtained by comparing this empirical distribution to the distribution for height associated variants. Since there are many height associated variants across the genome, this resampling procedure may bias to the null hypothesis, which can be conservative. A similar resampling procedure was used as we previously described.

## CARe

The authors wish to acknowledge the support of the National Heart, Lung, and Blood Institute and the contributions of the research institutions, study investigators, field staff and study participants in creating this resource for biomedical research. The following nine parent studies have contributed parent study data, ancillary study data, and DNA samples through the Broad Institute (N01-HC-65226) to create this genotype/phenotype data base for wide dissemination to the biomedical research community:

### Atherosclerotic Risk in Communities (ARIC)

The Atherosclerosis Risk in Communities Study is carried out as a collaborative study supported by the National Heart, Lung, and Blood Institute contracts N01-HC-55015, N01-HC-55016, N01-HC-55018, N01-HC-55019, N01-HC-55020, N01-HC-55021 and N01-HC-55022, and grants R01HL087641, R01HL59367, R37HL051021, R01HL086694 and U10HL054512; National Human Genome Research Institute contract U01HG004402; and National Institutes of Health contract HHSN268200625226C. Infrastructure was partly supported by Grant Number UL1RR025005, a component of the National Institutes of Health and NIH Roadmap for Medical Research; Cleveland Family Study (CFS): Case Western Reserve University (RO1 HL46380-01-16); Coronary Artery Risk in Young Adults (CARDIA): University of Alabama at Birmingham (N01-HC-48047), University of Minnesota (N01-HC-48048), Northwestern University (N01-HC-48049), Kaiser Foundation Research Institute (N01-HC-48050), University of Alabama at Birmingham (N01-HC-95095), Tufts-New England Medical Center (N01-HC-45204), Wake Forest University (N01-HC-45205), Harbor-UCLA Research and Education Institute (N01-HC-05187), University of California, Irvine (N01-HC-45134, N01-HC-95100); **J**ackson Heart Study (JHS): Jackson State University (N01-HC-95170), University of Mississippi (N01-HC-95171), Tougaloo College (N01-HC-95172); Multi-Ethnic Study of Atherosclerosis (MESA): University of Washington (N01-HC-95159), Regents of the University of California (N01-HC-95160), Columbia University (N01-HC-95161), Johns Hopkins University (N01-HC-95162), University of Minnesota (N01-HC-95163), Northwestern University (N01-HC-95164), Wake Forest University (N01-HC-95165), University of Vermont (N01-HC-95166), New England Medical Center (N01-HC-95167), Johns Hopkins University (N01-HC-95168),Harbor-UCLA Research and Education Institute (N01-HC-95169).

## Electronic supplementary material

Supplementary Figures
