# Supplementary material for: Height associated variants demonstrate assortative mating in human populations
Source: Sci Rep. 2017 Nov 16;7:15689. doi: 10.1038/s41598-017-15864-x (PMC5691191; doi:10.1038/s41598-017-15864-x)

Supplementary Information

Height associated variants demonstrate assortative mating in human populations

Xiaoyin Li[1], Susan Redline[2], Xiang Zhang[3], Scott Williams[1], Xiaofeng Zhu[1*]

[1] Department of Population and Quantitative Health Sciences, School of Medicine, Case Western Reserve University, Cleveland, OH, 44106, USA;

[2] Departments of Medicine, Brigham and Women's Hospital and Beth Israel Deaconess Medical Center, Harvard Medical School, Boston, MA, USA;

[3] College of Information Sciences and Technology, Pennsylvania State University, University Park, PA, USA.

[*] corresponding author: Xiaofeng Zhu, PhD, Department of Population and Quantitative Health Sciences, School of Medicine, Case Western Reserve University, Room 1317, Wolstein Research Building, 2103 Cornell Rd, Cleveland, OH 44106. Tel:216.368.0201. Email: xiaofeng.zhu@case.edu. Fax: 216-368-4880.

Supplementary Table S1 Inbreeding coefficients estimated using the height associated variants and frequency matched random variants in individuals after excluding outliers according the first two PCs in ARIC European Cohort (there are N=6,432 subjects after exclusion).

Single locus analysis				***Multi-locus analysis***			***# snp***
Mean $\hat{f}$ (sd) of height-associated SNPs	***Mean $\hat{f}$ (sd) of frequency matched SNPs***	***P-value KS-test****	***P-value T-test***	***$\widehat{f_M}$ (sd)***	***average $\widehat{f_M}$ of frequency matched variants (sd)***	***P-value****	
-9.24×10^{-04} (1.7×10^{-02})	-2.286×10^{-03} (1.7×10^{-02})	***0.713***	***0.172***	-9.1×10^{-04} (5.4×10^{-04})	-1.998×10^{-03} (6.565×10^{-04})	0.021	***521***
6.91×10^{-04} (1.7×10^{-02})	-1.262×10^{-03} (1.7×10^{-02})	***0.0527***	***0.0128***	7.09×10^{-04} (2.6×10^{-04})	-1.955×10^{-03} (2.877×10^{-04})	***0.001***	***2,500***
6.63×10^{-04} (1.7×10^{-02})	-1.639×10^{-03} (1.7×10^{-02})	***1.44*** $\times 10^{-08}$	***8.58*** $\times 10^{-14}$	6.99×10^{-04} (1.9×10^{-04})	-1.939×10^{-03} (1.67×10^{-04})	***0.001***	***5,000***

* Kolmogorov–Smirnov test

******P-value is comparing $\widehat{f_M}$ using height variants and randomly sampled frequency matched variants.

sd – standard deviation

Supplementary Table S2 Comparison of inbreeding coefficient estimated from height associated variants with whole genome at single locus level

Populations		***Height-associated SNPs***		***Whole genome SNPs***		***P-value***		***Sample size***
		Mean $\hat{f}$ (sd)	***# snp***	***Mean $\hat{f}$ (sd)***	***# snp***	***KS-test****	***T-test***	
European American	ARIC	-1.137×10^{-03} (1.7×10^{-02})	521	-1.9×10^{-03} (1.48×10^{-02})	68,423	2.19×10^{-01}	1.4×10^{-01}	6,787
	CFS	4.173×10^{-03} (7.6×10^{-02})	595	-5.52×10^{-03} (7.66×10^{-02})	64,749	4.58×10^{-03}	2.08×10^{-03}	171
African American	CARDIA	9.764×10^{-03} (6.6×10^{-02})	158	-3.23×10^{-03} (3.16×10^{-02})	139,703	3.16×10^{-02}	1.39×10^{-02}	828
	MESA	1.276×10^{-02} (9.4×10^{-02})	168	5.79×10^{-04} (3.56×10^{-02})	141,317	3.56×10^{-02}	3.41×10^{-02}	1,147
	JHS	1.22×10^{-02} (6.7×10^{-02})	165	-3.6×10^{-04} (2.41×10^{-02})	141,484	2.41×10^{-02}	1.71×10^{-02}	941
	CFS	2.414×10^{-02} (1.16×10^{-02})	166	-9.46×10^{-03} (9.19×10^{-02})	119,600	1.12×10^{-05}	2.56×10^{-04}	121
	ARIC	8.4687×10^{-03} (6.1×10^{-02})	159	-2.17×10^{-03} (2.89×10^{-02})	139,239	2.74×10^{-01}	2.98×10^{-02}	1,504

* Kolmogorov–Smirnov test

sd – standard deviation

ARIC - Atherosclerosis Risk in Communities; CFS - Cleveland Family Study; CARDIA - Coronary Artery Risk Development in Young Adults; JHS - Jackson Heart Study;

Supplementary Table S3 Comparison of inbreeding coefficient estimated from height associated variants with randomly sampled frequency matched variants after LD pruning not based on minor allele frequency at single locus level

Populations		*Height-associated SNPs*		*Randomly sampled Frequency matched SNPs*		*P-value*		*Sample size*
		Mean $\hat{f}$ (sd)	***# snp***	***Mean $\hat{f}$ (sd)***	***# snp available for resampling***	***KS-test****	***T-test***	
European American	ARIC	-1.137×10^{-03} (1.7×10^{-02})	521	-2.33×10^{-03} (1.51×10^{-02})	108,919	1.58×10^{-01}	2.36×10^{-01}	6,787
		6.02×10^{-04} (1.4×10^{-02})	2,500	-2.36×10^{-03} (1.36×10^{-02})		7.39×10^{-06}	3.61×10^{-07}	
		6.5×10^{-04} (1.4×10^{-02})	5,000	-1.84×10^{-03} (1.51×10^{-02})		1.33×10^{-12}	9.80×10^{-16}	
	CFS	4.173×10^{-03} (7.6×10^{-02})	595	-4.61×10^{-03} (7.61×10^{-02})	107,923	7.73×10^{-10}	5.13×10^{-03}	171
African American	CARDIA	9.764×10^{-03} (6.6×10^{-02})	158	-2.23×10^{-03} (4.01×10^{-02})	192,352	4.06×10^{-03}	2.29×10^{-02}	828
	MESA	1.276×10^{-02} (9.4×10^{-02})	168	2.85×10^{-04} (3.14×10^{-02})	192,843	6.55×10^{-03}	3.01×10^{-02}	1,147
	JHS	1.22×10^{-02} (6.7×10^{-02})	165	-5.26×10^{-04} (3.48×10^{-02})	193,613	5.6×10^{-04}	1.57×10^{-02}	941
	CFS	2.414×10^{-02} (1.16×10^{-02})	166	-8.81×10^{-03} (9.0×10^{-02})	174,979	3.71×10^{-08}	3.33×10^{-04}	121
	ARIC	8.4687×10^{-03} (6.1×10^{-02})	159	-1.66×10^{-03} (2.85×10^{-02})	199,605	1.77×10^{-01}	3.85×10^{-02}	1,504

* Kolmogorov–Smirnov test

sd – standard deviation

ARIC - Atherosclerosis Risk in Communities; CFS - Cleveland Family Study; CARDIA - Coronary Artery Risk Development in Young Adults; JHS - Jackson Heart Study;

Supplementary Fig. S1 The distribution of inbreeding coefficients for height variants (right) and for the whole genome (left). (a) ARIC (European); (b): CFS (European)

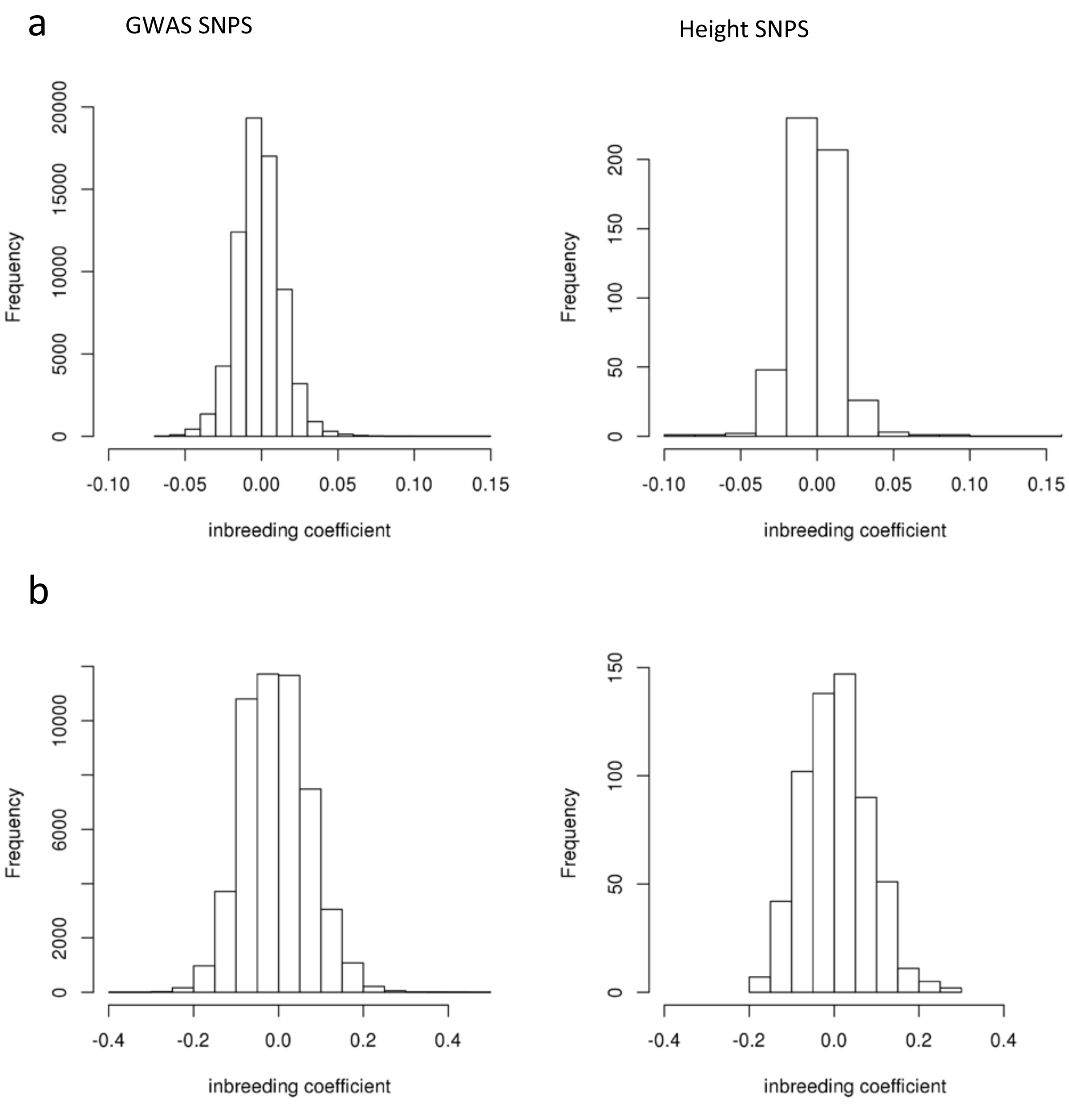

Supplementary Fig. S2 The distribution of inbreeding coefficients for height variants (right) and for the whole genome (left). (a) ARIC (African); (b) CARDIA (African); (c): CFS (African)

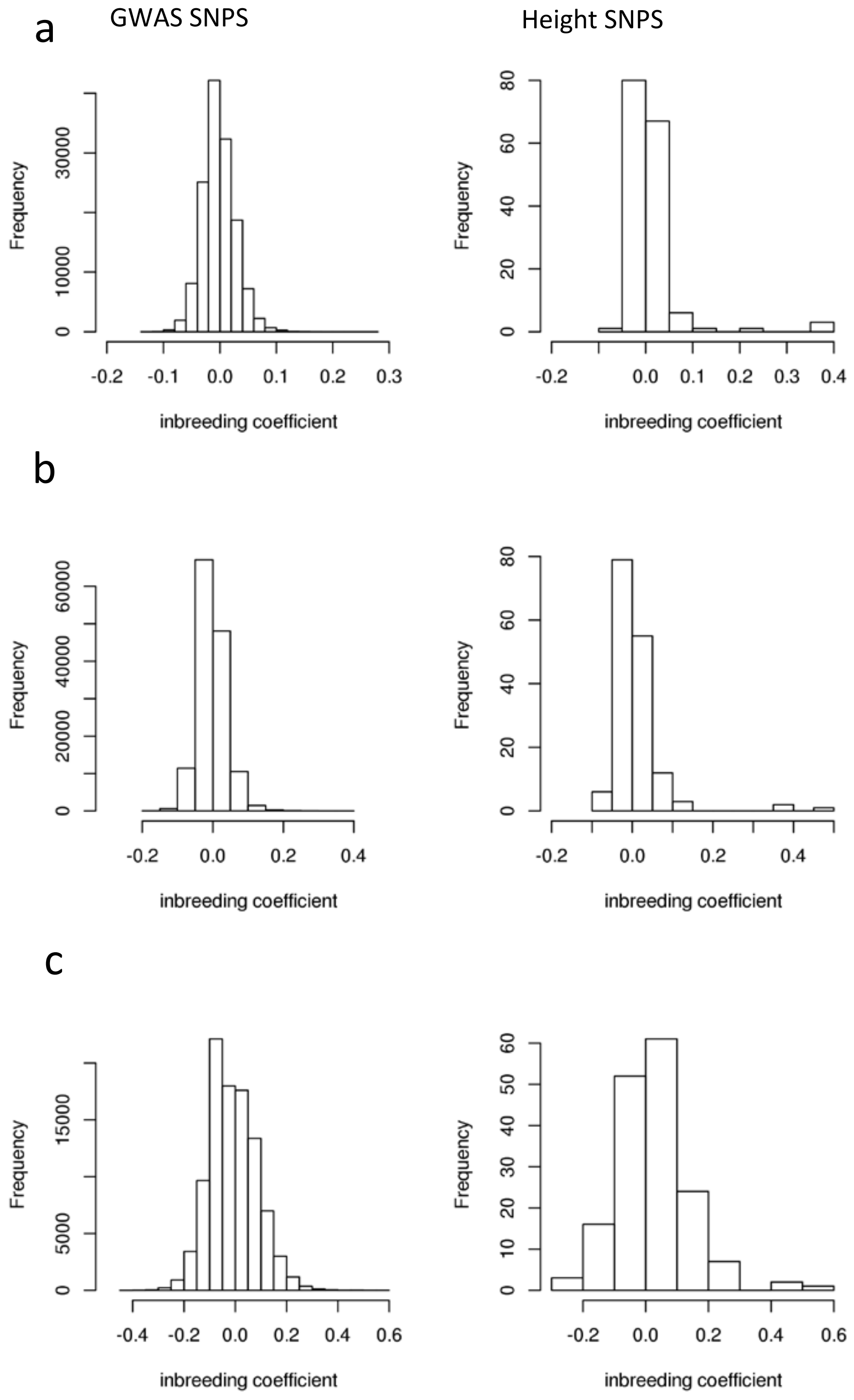

Supplementary Fig. S3 The distribution of inbreeding coefficients for height variants (right) and for the whole genome (left). (a) JFS (African); (b) MESA (African)

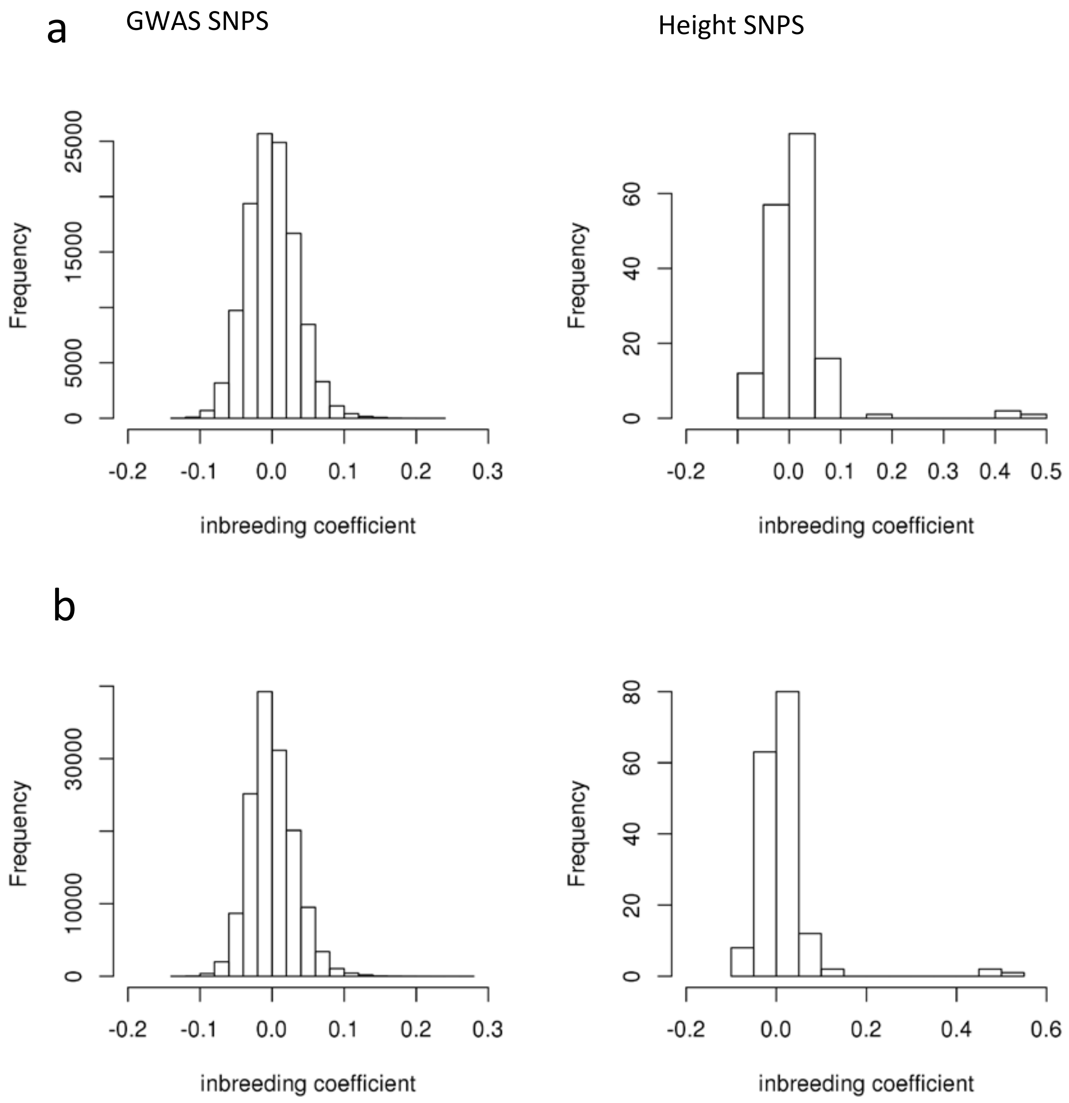

Supplementary Fig. S4 Principal components analyses. (a): CFS (European); (b) CARDIA (African); (c) MESA (African); (d): JHS(African); (e) CFS (African); (f) ARIC (African)

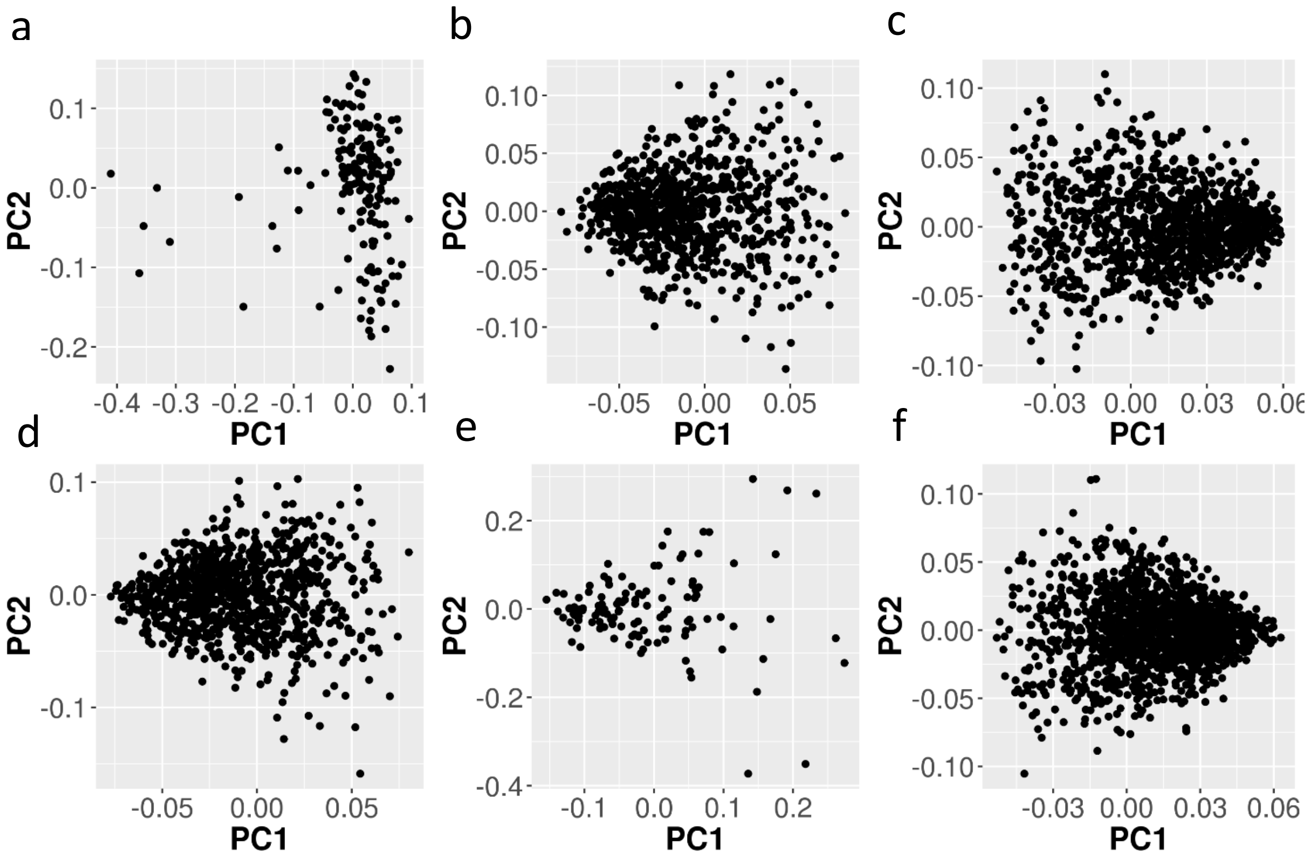

Supplementary Fig. S5 Minor allele frequency distribution for height variants and for the whole genome. (a) ARIC (European); (b): CFS (European)

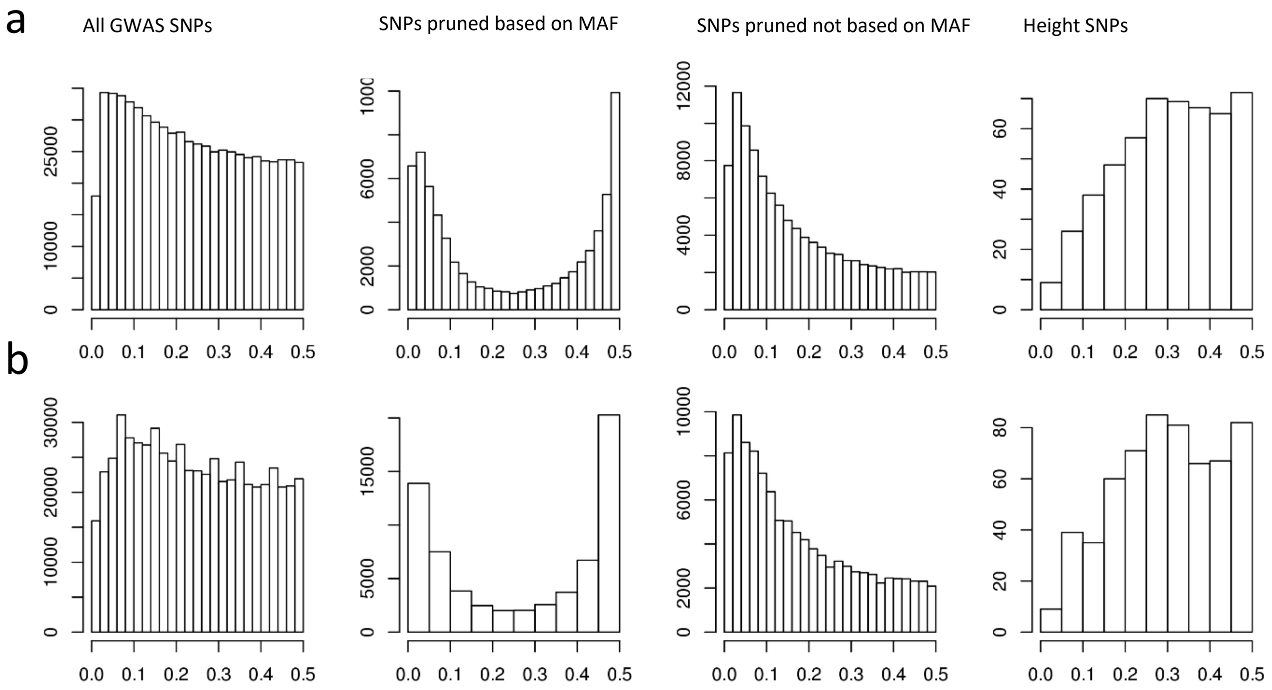

Supplementary Fig. S6 Minor allele frequency distribution for height variants and for the whole genome. (a) ARIC (African); (b) CARDIA (African); (c) CFS (African); (d) JHS (African); (e) MESA (African)

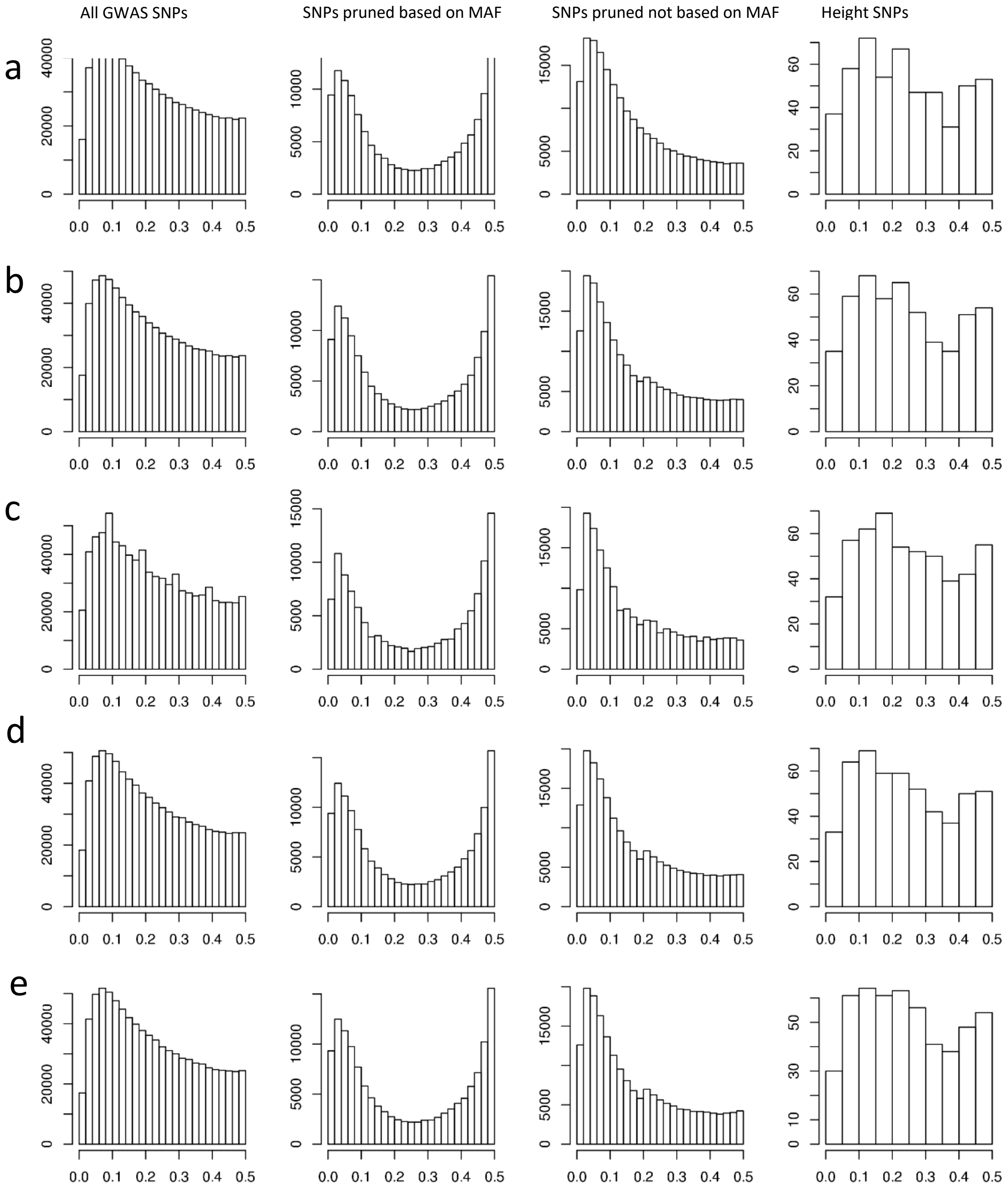

Supplement: Supplementary file 1 — Supplementary Figures [file 41598_2017_15864_MOESM1_ESM.pdf]
